# Adaptive value of positive illusions: coping with adversity and improving relationships

**DOI:** 10.3389/fpsyg.2025.1540566

**Published:** 2025-08-06

**Authors:** Hongyan Chen

**Affiliations:** School of Psychology, Henan Medical University, Xinxiang, China

**Keywords:** positive illusion, adaption, adversity response, relationship improvement, happiness

## Abstract

The concept of positive illusions is rooted in positive psychology, and is essential for maintaining individual physical and mental health. This study primarily discusses the adaptive value of positive illusions from two perspectives: adversity response and relationship improvement. As a positive cognition, positive illusions can promote the fight against disease and accelerate the psychological recovery after traumatic events. They can also beautify the impression of others through positive projection, thereby helping in establishing positive, stable, and harmonious interpersonal relationships. Future research should expand the adaptive value of positive illusions in practical domains, exploring their positive roles in occupational contexts, clinical interventions, and parent–child relationships, while deepening investigations into their underlying brain mechanisms.

## 1 Introduction

Before the 1970s and 1980s, most psychologists held the view that an objective, true, and correct self-perception was the only way to achieve happiness and success. In other words, Well-adjusted individuals possess complete and accurate self-perception, while those with persistent misconceptions about themselves and reality are considered maladjusted. This may not be entirely correct. Taylor and Brown (1988) originally conceptualized positive illusions as a form of cognitive filtering and adaptive psychological adjustment, manifested when individuals perceive themselves, their current reality, or future prospects in daily life or under stressful circumstances. They posited that most mentally healthy individuals exhibit systematic positive biases. Positive illusion differs from the concept of self-enhancement. Such positive perceptions included positive self-evaluations, exaggerated perception of ability to control events, and tendency to be unrealistically optimistic toward the future. Positive illusions are conceptually distinct from self-enhancement. Compared to positive illusion, self-enhancement encompasses a broader scope and manifests in more diverse forms; positive illusion is merely a typical manifestation of self-enhancement. Additionally, self-enhancement pertains solely to an individual’s self-improvement, whereas positive illusion exists not only in an individual’s perception of themselves but also in their perception of significant others.

This positive cognitive bias contributed to their adaptation, as it helped them in protecting self-esteem, promote physical and mental health, and maintain happiness (Taylor and Brown, 1988). In recent years, human society has faced unprecedented challenges and adversities, such as the global public health crisis (e.g., the COVID-19 pandemic), the increasingly severe impacts of climate change and widespread socio-economic uncertainties. These persistent stressors pose a severe test to the mental health and resilience of both individuals and communities. In the current context, re-examining the adaptive value of positive illusions holds unprecedented urgency and importance.

Researchers hold differing views on the relationship between positive illusions and adaptation. Some critics of Taylor and Brown’s theory argue that excessive positive cognition can lead to adverse outcomes. For instance, heightened positive illusions may increase aggressive behaviors because individuals, holding inflated self-views, may react impulsively with aggression when their positive self-concept is threatened by the environment, due to difficulties in accepting reality (Baumeister et al., 1996). Furthermore, an exaggerated sense of control can lead people to engage in risky behaviors such as dangerous driving, reckless investing, and alcohol abuse (Hablemitoğlu and Yıldırım, 2008). However, researchers who hold critical views toward positive illusions neglect the original intent behind Taylor and Brown’s proposal of this concept and theoretical framework. Extremely exaggerated self-awareness and sense of control are equivalent to arrogance, which is not conducive to individual adaptation. It is crucial to distinguish between adaptive positive illusions and severely distorted self-aggrandizement. Research by Taylor et al. (2000) and Dufner et al. (2019) indicates that the effects of positive illusions follow an inverted U-shaped curve. Its peak depends on the level of environmental threat and the individual’s threshold of psychological resilience, meaning that self-enhancement is more applicable in high-stress situations, and individuals with low self-esteem benefit more easily from positive illusions. Furthermore, from the perspective of cognitive mechanisms, the motivated reasoning model suggests that the essence of positive illusions is goal-directed cognitive processing. Individuals engage in selective information processing to maintain self-esteem, with its intensity being modulated by motivational needs (Kunda, 1990). Dual-process theory posits that the adaptiveness of positive illusions depends on the dynamic interaction between the automatic system (which rapidly activates positive self-schemas) and the reflective system (which conducts evidence-based self-evaluation) (Strack and Deutsch, 2004). From this perspective, the purpose of positive illusions is to protect self-esteem and to attain and enhance psychological well-being, thus serving as a form of positive psychological adaptation.

Taylor and Brown (1994) also noted that only a small minority of people exhibit extreme narcissism or excessively inflated self-views. Generally, the positive illusions held by mentally healthy individuals are moderate rather than extreme. Therefore, positive illusions generally serve an adaptive function. This article primarily examines the practical value of positive illusions from two perspectives: coping with adversity and improving relationships, then proposes future research directions.

## 2 Theoretical origin and adaptive value of positive illusions

### 2.1 The need for human evolution

Evolutionary psychologists have highlighted that the mind and behaviors that human beings now possess usually have important adaptive value in the evolutionary process, which can directly or indirectly promote the reproduction of races. Only individuals with positive optimism about reality have the courage and motivation to overcome the dangers and threats to reproduction. From the perspective of evolution theory, a positive illusion is a characteristic that humans have retained in the process of species evolution, and this cognitive bias, which is conducive to human survival and reproduction, has been internalized into human genes. Moreover, this positive self-perception begins early in life.

Johnson et al. (2011) proposed an evolutionary model of positive illusions based on Taylor’s research on positive illusions, combined with game theory and evolutionary psychology. The model suggests that throughout human history, positive illusions provided evolutionary advantages. By overestimating their abilities, individuals gained ambition and persistence, enhancing resource acquisition in competitive environments. This increased survival odds and reproductive success through natural selection.

### 2.2 Adaptive representations of cognitive system

Cognitive Adaptation Theory posits that positive illusions stem from the brain’s selective filtering of information, whereby individuals adjust their interaction with the environment through cognitive processes to achieve adaptive goals of survival and development. Crucially, these positive cognitive biases do not represent cognitive defects but rather represent adaptive strategies shaped by evolutionary processes, designed to strengthen psychological resilience and behavioral motivation. By employing mechanisms such as selective attention, biased encoding, and memory reconstruction, individuals screen and restructure environmental information to reduce cognitive load and enhance coping efficiency. Furthermore, the cognitive system actively adjusts its mental representations of reality based on both short-term survival demands (e.g., stress responses) and long-term developmental objectives (e.g., self-actualization).

### 2.3 The motivational mechanism of self-determination

Self-Determination Theory provides crucial theoretical support for the adaptive function of positive illusions. This theory emphasizes that when individuals satisfy their three fundamental psychological needs—autonomy, competence, and relatedness—through positive illusions (such as overestimating personal abilities, exaggerating perceived control over the environment, or idealizing interpersonal relationships), deeper adaptive mechanisms are activated. Specifically: Positive illusions can reinforce the belief that “I can master adversity,” thereby preserving a sense of control in decision-making during difficult times; Individuals with positive illusions directly enhance their efficacy beliefs in facing challenges through a moderate overestimation of their own capabilities; Furthermore, positive illusions between partners or between parents and children can strengthen social bonds by enhancing relationship satisfaction. In the process of fulfilling psychological needs, positive illusions serve as a motivational resource integration strategy, enabling individuals to maintain psychological equilibrium while continuously gaining momentum for growth.

### 2.4 The need for self-realization

Humanistic psychologist Carl Rogers posited that humans possess an innate actualizing tendency—a motivation toward growth and self-actualization. This inherent drive propels individuals “toward positivity and constructiveness,” ultimately enabling the maximal realization of their potential. The actualizing tendency is a fundamental biological inclination, present in all organisms, to strive toward maintaining, enhancing, and ultimately actualizing themselves. Achieving self-actualization requires moving in directions that fully develop individual potential, which inherently involves constructive and creative pursuits. In this sense, individuals’ positive illusions about themselves and the external world represent an expression of this actualizing tendency.

## 3 Positive illusions and individual adaptation

### 3.1 Positive illusions and adversity response

Positive illusions foster psychological resilience by reframing adversity cognition, transforming threatening appraisals into growth-oriented interpretations, thereby empowering individuals to confront setbacks and disasters with enhanced confidence and courage. First, positive illusions play a vital role in coping with illness. As a positive cognitive style, they exert beneficial effects by delaying disease deterioration, enhancing treatment adherence, and promoting recovery. These illusions provide essential psychological resources that support longevity and health maintenance. Taylor et al. (2000) found that breast cancer and HIV/AIDS patients who maintained positive illusions of control over their illness fostered a positive psychological environment. This helped sustain hope and motivated proactive disease management. For instance, breast cancer patients with stronger positive illusions often believed they would remain cancer-free, adopting more adaptive coping strategies. Conversely, patients perceiving loss of control were more prone to depression, defeatism, or psychological collapse. Supporting this, Affleck et al.’s (1987) tracking study of male heart attack survivors revealed that those with positive illusions about their cardiac event had a reduced risk of recurrence over the subsequent 8 years. Schiavon et al. (2013) found that positive survival expectations among cancer patients were associated with lower depressive symptoms.

Furthermore, positive illusions are crucial for coping with stressful and traumatic events, significantly reducing stress and accelerating psychological recovery. Taylor et al. (2003) examined whether positive illusions confer a protective biological function under stress. Participants with or without positive illusions performed stressful tasks (e.g., serial subtraction); those with positive illusions exhibited healthier biological responses, including lower baseline cortisol and reduced autonomic activation during stress. Similarly, Bonanno et al. (2005) found that individuals with positive illusions directly exposed to the 9/11 attacks recovered faster and demonstrated better post-disaster adaptation. Van and Nowak (2011) noted that positive illusions effectively dampen physiological stress responses (e.g., reduced cardiovascular reactivity), facilitated by positive emotions, constructive coping, and social support. Mazur et al. (1999) also demonstrated that positive illusions mitigate negative effects and enhance adjustment in children (aged 9–12) from divorced families.

Therefore, When confronting adversity, positive illusions confer adaptive value through three integrated mechanisms: cognitively reframing threats as manageable challenges to reduce perceived uncontrollability; affectively sustaining positive emotional states that buffer stress hormone responses and enhance psychological resilience; behaviorally activating goal-directed actions by mobilizing psychosomatic resources through optimistic expectations. This represents a coordinated cognitive-affective-behavioral defense system: subjectively minimizing threat severity preserves mental energy for coping, while objectively driving adaptive engagement to transform potential trauma into growth opportunities.

### 3.2 Positive illusions and relationship improvement

Brown and Taylor (1986) noted that positive illusions extend beyond self-perception to intimate others. Building on interdependence theory, Murray et al. (1996) proposed that individuals form idealized impressions of partners through projection—projecting their ideals onto actual partners and perceiving flaws positively. This positive illusion plays an important role in improving relationships and enhancing relationship satisfaction.

As the folk saying “Beauty is in the eye of the beholder” goes, positive illusions operate with particular potency in enhancing romantic and marital relationships. Barelds et al. (2011) found that romantic partners exhibit positive illusions in assessing their own and their partner’s body attractiveness, enhancing relationship stability. Moreover, Leo (2014) demonstrated that such illusions increase tolerance for partner faults. He (2017) showed that positive illusions in evaluating a partner’s appearance and personality correlate with higher relationship satisfaction and longitudinally predict this satisfaction.

In marriage, partners often fear their spouse may be less than ideal. When confronting negative aspects or conflicts, they restore cognitive equilibrium by exaggerating the partner’s positive traits, downplaying shortcomings, and projecting ideal selves onto them to preserve their own sense of security. Barelds-Dijkstra and Barelds (2008) studied 93 couples married 14+ years, finding partners rated their spouse’s physical attractiveness higher than their own. This positive illusion enhanced intimacy and satisfaction. Similarly, Barelds and Dijkstra (2009) found positive illusions about a partner’s physical attractiveness increased relationship satisfaction in 177 Dutch heterosexual couples, particularly pronounced in older couples. Engdahl (2015) also confirmed that such illusions promote marital satisfaction.

Murray et al. (1996) suggested that positive illusions not only existed in love and marriage but also existed between parents and children. As biological extensions of the self, children elicit inherent parental self-enhancement tendencies, resulting in positively biased evaluations. Culturally embedded aspirations (“expecting sons to become dragons, daughters phoenixes”) further drive unrealistic positive cognitions. Blood-bonded emotional ties then trigger involuntary idealization. Wenger and Fowers (2008) found parents of preschoolers typically perceive their children as having more positive and fewer negative traits than average.

Therefore, positive illusions enhance intimate relationships through three integrated mechanisms: cognitively, they reshape perception by selectively amplifying partner virtues while minimizing flaws, reducing conflict attribution bias; affectively, sustained positive emotions buffer relational friction and accelerate post-conflict recovery through heightened resilience; behaviorally, idealized perceptions motivate prosocial investments (e.g., accommodation, sacrifice), which elicit reciprocal partner responses to form a self-reinforcing cycle of cognitive optimization, behavioral reinforcement, and relational improvement. Essentially, this psychological system lubricates relationship maintenance by lowering cognitive load, boosting emotional security, and fostering behavioral reciprocity, thereby dynamically converting subjective cognitive bias into objective relational gains.

### 3.3 Positive illusions and individual happiness

Positive illusions promote enhanced well-being through self-enhancement, relational enhancement, and proactive coping mechanisms. Marshall and Brown (2008) establish that positive illusions foster subjective well-being by generating positive affect, enhancing relationships, and improving coping efficacy. These effects indirectly strengthen psychological resilience and adaptability, facilitating holistic growth that elevates well-being.

Jiang et al. (2015) revealed that these illusions predict self-harmony levels, as individuals maintain idealized future expectations and optimism, thereby enhancing self-acceptance. Similarly, Zheng (2017) indicated that positive illusions can enhance an individual’s self-confidence and self-efficacy, motivating them to more proactively confront and resolve difficulties, thereby improving life satisfaction. Therefore, positive illusions generate beneficial psychological experiences, facilitating a state of psychological fluency that enhances mental well-being.

Across adversity coping and relationship enhancement, positive illusions recalibrate self-perception to interpret reality through optimally framed possibilities. This fosters inner harmony, stable relationships, and heightened well-being and meaning—constituting an adaptive form of life wisdom.

## 4 Future research directions

With the rise of contemporary positive psychology, the adaptive value of positive illusions has been attracting increasing attention. Future research in this area can be expanded in the following aspects.

### 4.1 Deepen the research on the adaptive value of positive illusions in individuals’ work fields

Extensive research confirms the adaptive value of positive illusions during stressful or threatening events. Additionally, studies demonstrate their benefits in daily work domains. Anastasia (2016) found they enhance task persistence and sustain motivation, improving work efficiency. Ottesen and Gronhaug (2005) showed that positive illusions are crucial for workplace innovation, fostering greater challenge-seeking, optimism, and confidence when facing difficult tasks, thereby maximizing potential. Stankov and Lee (2008) revealed that positive illusions facilitate creative thinking by protecting self-esteem through selective memory, simplifying cognition via positive affect, and focusing attention on successes, ultimately fostering innovation. Current research on workplace positive illusions predominantly relies on samples from Western individualistic cultures, while studies in collectivist cultural contexts remain scarce. It is therefore imperative to strengthen cross-cultural comparative investigations in future research.

### 4.2 Expanding the practical study on the adaptive value of positive illusions in parent–child relationships

A large number of studies on positive illusions in marriage and love relationships have confirmed that positive illusions play a positive role in improving relationship satisfaction between lovers and couples, while discussions on positive illusions in parent–child relationships are still relatively lacking at home and abroad. Existing research has predominantly concentrated on parents’ positive illusions toward their children, while neglecting investigations into offspring’s idealized perceptions of parents and their crucial bidirectional adaptive value. Future research necessitates robust investigation of the adaptive functions of mutual positive illusions within parent–child dyads.

### 4.3 Exploring the clinical application value of positive illusions

Early research indicates depressed patients exhibit lower perceived personal control compared to non-depressed individuals, reflecting characteristic depressive cognition. Implementing positive illusion interventions may enhance personal control in depression, potentially alleviating symptoms.

Moreover, positive illusions critically aid stress coping and reduction (Taylor et al., 2003). For stress-related conditions (e.g., IBS, immune disorders), combining positive illusion interventions with mindfulness practices could examine their clinical efficacy.

### 4.4 Focus on positive illusions’ brain mechanisms and cross-cultural study

From an evolutionary perspective, positive illusions represent an adaptive trait preserved through human evolution. As a manifestation of positive illusions, optimism bias has been widely investigated by cognitive neuroscientists using fMRI to elucidate its neural bases. Several studies have shown that the neural correlates of optimism bias are present in the amygdala, anterior rostral cingulate cortex, prefrontal cortex, and dopaminergic pathways (Blair et al., 2013; Hughes and Beer, 2012; Eisenberger et al., 2011; Kawamoto et al., 2012; Kross et al., 2011; Meyer et al., 2011; Schacter and Addis, 2007; Sharot et al., 2012). Sharot et al. (2011) demonstrated through fMRI that the rostral anterior cingulate cortex (rACC) and amygdala show significant activation when processing positive future events, while simultaneously suppressing pessimistic information. This neural bias causes approximately 80% of individuals to systematically overestimate the probability of favorable outcomes.

Sasaki et al. (2011) revealed a gene–environment interaction wherein carriers of the 5-HTTLPR short allele exhibited a 300% greater buffering effect of positive illusions against depression in supportive environments, demonstrating how neurogenetic mechanisms modulate the adaptive value of cognitive biases. Studies on the neural mechanisms of optimism bias have shown that the brain selectively processes positive information through the anterior rostral cingulate cortex-amygdala pathway, which provides a biomechanical explanation for how positive illusions enhance the ability to cope with adversities.

Cross-cultural evidence reveals culturally distinct self-enhancement patterns: Westerners favor explicit self-promotion, while collectivists use implicit strategies like in-group comparisons to sustain positive illusions, demonstrating cultural moderation of self-enhancement’s adaptive function (Dufner et al., 2019). Future brain mechanism research on the adaptive value of positive illusions should focus on dynamic regulation of neural circuits, gene-neuro-environment interactions, and multimodal modeling; Meanwhile, cross-cultural studies should explore neural plasticity of cultural values, customized interventions, and methodological innovation.
